# The Antimethanogenic Nitrocompounds Can be Cleaved into Nitrite by Rumen Microorganisms: A Comparison of Nitroethane, 2-Nitroethanol, and 2-Nitro-1-propanol

**DOI:** 10.3390/metabo10010015

**Published:** 2019-12-25

**Authors:** Zhen-Wei Zhang, Yan-Lu Wang, Wei-Kang Wang, Yong-Yang Chen, Xue-Meng Si, Ya-Jing Wang, Wei Wang, Zhi-Jun Cao, Sheng-Li Li, Hong-Jian Yang

**Affiliations:** State Key Laboratory of Animal Nutrition, College of Animal Science and Technology, China Agricultural University, Beijing 100193, China; qingyibushuo@163.com (Z.-W.Z.); yanluwang@yeah.net (Y.-L.W.); 18292092306@163.com (W.-K.W.); yanz00@foxmail.com (Y.-Y.C.); sxmswun@126.com (X.-M.S.); yajingwang@cau.edu.cn (Y.-J.W.); wei.wang@cau.edu.cn (W.W.); caozhijun@cau.edu.cn (Z.-J.C.); lisheng0677@163.com (S.-L.L.)

**Keywords:** nitroethane, 2-nitroethanol, 2-nitro-1-propanol, rumen microorganism, degradation, metabolism, in vitro

## Abstract

A class of aliphatic short chain nitrocompounds have been reported as being capable of CH_4_ reduction both in vitro and in vivo. However, the laboratory evidence associated with the metabolic fate of nitrocompounds in the rumen has not been well documented. The present study was conducted to compare in vitro degradation and metabolism of nitroethane (NE), 2-nitroethanol (NEOH), and 2-nitro-1-propanol (NPOH) incubated with mixed rumen microorganisms of dairy cows. After 10 mM supplementation of nitrocompounds, a serious of batch cultures were carried out for 120 h under the presence of two substrates differing in the ratio of maize meal to alfalfa hay (HF, 1:4; LF, 4:1). Compared to the control, methane production was reduced by 59% in NPOH and by >97% in both NE and NEOH, and such antimethanogenic effects were more pronounced in the LF than the HF group. Although NE, NEOH, and NPOH addition did not alter total VFA production, the rumen fermentation pattern shifted toward increasing propionate and butyrate and decreasing acetate production. The kinetic disappearance of each nitrocompound was well fitted to the one-compartment model, and the disappearance rate (*k*, %/h) of NE was 2.6 to 5.2 times greater than those of NEOH and NPOH. Higher intermediates of nitrite occurred in NEOH in comparison with NPOH and NE while ammonia N production was lowest in NEOH. Consequently, a stepwise accumulation of bacterial crude protein (BCP) in response to the nitrocompound addition was observed in both the HF and LF group. In brief, both NE and NEOH in comparison with NPOH presented greater antimethanogenic activity via the shift of rumen fermentation. In addition, the present study provided the first direct evidence that rumen microbes were able to cleave these nitrocompounds into nitrite, and the subsequent metabolism of nitrite into ammonia N may enhance the growth of rumen microbes or promote microbial activities.

## 1. Introduction

Aliphatic nitrocompounds consist of a straight-chained carbon backbone having one or more nitro and hydroxyl or carboxyl functional groups. Naturally occurring 3-nitropropanol and 3-nitropropionic acid were found in some rangeland plants (e.g., *Astragalus*, *Coronilla*, and *Indigofera*) [1]. Additionally, nitroethane (NE), 2-nitroethanol (NEOH), and 2-nitro-1-propanol (NPOH) have been produced as industrial chemicals and synthetic intermediates for years [2]. Meanwhile, the aforementioned nirocompounds were reported to present biological anti-pathogenic activity [3,4] and anti-methanogenic activity in ruminants [5,6,7,8]. The toxicity of nitrocompounds has been elucidated and reviewed in previous studies with chickens, mice, pigs, rabbits, and rats [1,9,10]. In contrast with previous studies in monogastric animals, Anderson et al. noted that rumen microbes were able to metabolize the naturally occurring 3-nitro-1-propionate and 3-nitro-1-propanol into β-alanine and 3-amino-1-propanol, respectively [11]. Except for that study, limited information is available about direct laboratory evidence associated with the possible metabolic fate of the aforementioned nitrocompounds in ruminal ecology. In this study, we hypothesized that rumen microorganisms could metabolize these nitrocompounds to degradable nitrite, and ammonia N generated from the metabolism of nitrocompounds could be partially utilized for synthesizing microbial amino acids during rumen fermentation. Thus, the objectives of the present study were twofold. Firstly, it compared the kinetic clearance rate of NE, NEOH, and NPOH incubated with mixed rumen microorganisms. Secondly, it examined the metabolic product of nitrite and ammonia N, and the latter was supposed to promote the growth of rumen microorganisms.

## 2. Results

### 2.1. Effect of Nitrocompounds on VFA Production and Fermentation Gas Pattern

Total VFA production was remarkably greater in low-forage substrate (LF) than high-forage HF) substrate (Table 1, *p* < 0.01), but it was not affected by the addition of nitrocompounds. Regarding the VFA patterns, the molar acetate proportion was higher in the HF than LF substrate while the molar percentages of propionate, butyrate, and branch-chained volatile fatty acid (BCVFA) were lower in HF than LF substrate (*p* < 0.01). As a result, the acetate:propionate ratio was higher in the HF than LF substrate (*p* < 0.01). Interaction between the substrate and nitrocompounds treatment did not occur for total VFA and individual VFAs. The supplementation of NE, NEOH, and NPOH in comparison with the controls decreased the molar acetate proportion, but they increased the molar percentage of propionate and butyrate, resulting in a decrease of the acetate:propionate ratio (*p* < 0.01).

As shown in Table 2, no interaction occurred for fermentation gas production between the substrate and nitrocompounds. Total gas production (GP_120_) was lower in HF than LF (*p* < 0.01). The GP_120_ was decreased in both NE and NEOH in comparison with the control (*p* < 0.01), but no difference occurred between NPOH and the control for HF substrate. CH_4_ production was remarkably decreased with NE, NEOH, and NPOH addition (*p* < 0.01), and such inhibition was more pronounced in the LF than HF substrate. Conversely, the nitrocompound addition increased the composition of both the H_2_ and CO_2_ content, and the molar proportion of H_2_ was obviously greater in the LF than the HF substrate (*p* < 0.01).

### 2.2. Disappearance Kinetics of NE, NEOH, and NPOH

**Figure 1:**
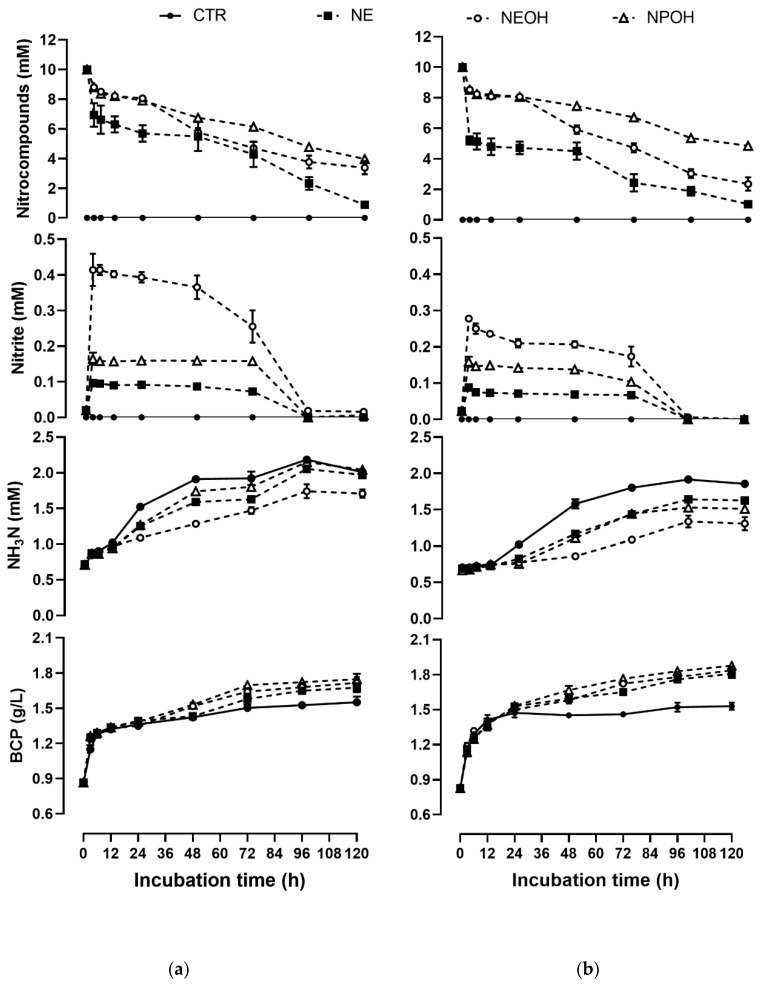
Variation of nitrocompounds, nitrite, ammonia N, and bacterial crude protein (BCP), on average, in culture fluids during in vitro ruminal fermentation of substrates with a high (HF, **a**) and low (LF, **b**) forage content incubated in batch cultures of mixed rumen microorganisms. CTR, control; NE, nitroethane; NEOH, 2-nitroethanol; NPOH, 2-nitropropional. Effect of treatments was significant at *p* < 0.01.

After the fixed amount of nitrocompounds were added in culture fluids, as expected, the residual content of nitrocompounds declined against the incubation time (Figure 1a,b). After 120 h incubation, 92% of NE, 69% of NEOH, and 64% NPOH was degraded (Figure 1a) in the HF substrate, and 91% of NE, 79% of NEOH, and 56% NPOH was degraded (Figure 1b) in the LF substrate.

The disappearance of nitrocompounds in the culture fluids against different incubation times was well fitted to the one-compartment model (r > 0.96). Regardless of what type of substrate was incubated, the disappearance rate (k, %/h) in the one-compartment model was ranked as: NE > NEOH > NPOH (Table 3, *p* < 0.01). The time (T_1/2_) when half of the initial nitrocompound inclusion (10 mM) had disappeared was ranked as: NPOH > NEOH > NE (*p* < 0.01).

### 2.3. Effect of Nitrocompounds on Nitrite Accumulation

Nitrite was not detected in the control group during 120 h of incubation, however, a sharp accumulation of nitrite occurred in the nitrocompound-treated groups from 0 to 3 h of incubation and then the residual content of nitrite decreased remarkably against the incubation time in both HF (Figure 1a)and LF substrate (Figure 1b). In the nitrocompound-treated groups, up to 0.09 to 0.10, 0.28 to 0.42, and 0.16 to 0.17 mM of nitrite was accumulated in the NE, NEOH, and NPOH group after 3 h of incubation, respectively. Subsequently, the nitrite was totally degraded in the nitrocompound-added cultures with both the HF and LF substrate. The maximum concentration of nitrite (C_3_) was ranked as: NEOH > NPOH > NE. Regarding the nitrite disappearance kinetics (Table 4), its disappearance rate (k, %/h) from 3 to 120 h in the one-compartment model was ranked as: NE > NEOH > NPOH (Table 4, *p* < 0.01). Conversely, the time (T_1/2_) when half of the nitrite had disappeared was ranked as: NPOH > NEOH > NE (*p* < 0.01).

### 2.4. Effect of Nitrocompounds on Ammonia N Production

In both the control and nitrocompound-treated cultures, the content of ammonia N improved with the incubation time increasing in both HF (Figure 1a) and LF substrate (Figure 1b). The yield of ammonia N in control groups was greater than that in the nitrocompound-treated groups during 120 h incubation for both the LF and HF substrate. The yield of ammonia N at 120 h in comparison with 3 h was increased by 139% to 159%, 114% to 148%, 77% to 122%, and 111% to 160% in CTR, NE, NEOH, and NPOH, respectively. The asymptotic ammonia N accumulation (A) in the control group was greater than that in the nitrocompound-treated groups during 120 h of incubation for both the LF and HF substrate (Table 5). In addition, the accumulation rate (k, %/h) of ammonia N in both the HF and LF substrate was ranked as: CTR > NE > NPOH > NEOH. Conversely, T_1/2_ was ranked as: NEOH > NPOH > NE > CTR (*p* < 0.01).

### 2.5. Effect of Nitrocompounds on BCP Production

Throughout the incubation period, the content of BCP in culture fluids was elevated significantly in both HF substrate (Figure 1a) and LF substrate (Figure 1b). The content of BCP in the nitrocompount-treated groups was apparently greater than that in the control group, and this stimulation effect was more pronounced in the LF substrate (Figure 1b) than that in the HF substrate (Figure 1a). After 120 h of incubation, 60% to 69%, 84% to 89%, 89% to 92%, and 92% to 97% of BCP was produced in the CTR, NE, NEOH, and NPOH groups, respectively. The accumulation rate (Table 6; k, %/h) of BCP was significantly higher in the nitrocompound-treated groups than that in the control, and there was no statistical difference among the NE, NEOH, and NPOH treatments. The T_1/2_ was greater in the control than that in the nitrocompound-treated groups (*p* < 0.01).

## 3. Discussion

### 3.1. Fermentation Characteristics and Antimethanogenic Activity of Nitrocompounds

Ruminal methanogenesis is an evolutionary adaptation that enables the rumen ecosystem to dispose of H_2_, ultimately, maintaining a low partial pressure of H_2_ (at approximately 0.1 kPa) in the unperturbed rumen [12]. However, excessive accumulation of H_2_ could cause a decrease in fermentation efficiency, which, as a consequence, would limit the availability of oxidized cofactors required for glycolysis in ruminants [5]. In order to compensate for the disruption of reducing equivalents flow to the methanogenesis, some CH_4_ inhibition strategies often cause an increase of more reduced VFA (e.g., propionate, butyrate) production and the concomitant decrease of acetate [5]. In accordance with this phenomenon, NE, NEOH, and NPOH addition decreased acetate content but increased the accumulation of propionate and butyrate in the current study. The positive effect of NE, NEOH, and NPOH on propionate and butyrate production suggested that a portion of the reduced equivalents spared from CH_4_ production appeared to have been used for the production of more reduced VFAs. Although considerable amounts of H_2_ could not be accounted for in reduced fermentation products (e.g., propionate, butyrate) produced in the nitrocompound-treated incubations, fermentation efficiencies were not compromised within nitrocompound-treated cultures (see Table 1). The obvious accumulation of H_2_ within the cultures supplemented with NE, NEOH, or NPOH indicated that microbial interspecies-hydrogen transfer had not been completely optimized. Taken together, CH_4_ production inhibited by nitrocompounds may result from a direct inhibition against the methanogens by likely precluding their ability to competitively consume reducing equivalents [13]. Reductants can sometimes accumulate as formate, lactate, and ethanol under an increased H_2_ atmosphere [5]; however, these products were not determined in the present study, and thus they cannot be excluded as a potential fate of some of the reducing equivalents. In addition, the reductants were possiblly consumed by anabolic processes, which included microbial cell growth, intracellular polyhydroxyalkanoate, or extracellular polysaccharide synthesis [5].

The antimethanogenic activity of nitrocompounds has been extensively documented in the last few decades [5,6,14]. In accordance with the previous study, the present CH_4_ production was dramatically reduced by up to 99%, 99%, and 59% with NE, NEOH, and NPOH addition, respectively, when compared with the control group. In addition, the detectable nitrite, a portion of metabolic products of NE, NEOH, and NPOH, was also a potent inhibitor of methanogenesis within the present in vitro fermentation. From the thermodynamic perspective, conversion of nitrite into ammonia is more favorable than the reduction of CO_2_ to CH_4_ [15].

### 3.2. Nitrocompounds’ Degradation and Metabolic Fate

With respect to ruminal metabolism of nitrocompounds, plants containing nitrotoxins from their glucose conjugates were first documented [11]. Worldwide, more than 450 species and varieties of leguminous *Astragalus* were discovered to contain the glycoside conjugates of naturally occurring 3-nitropropanol and 3-nitropropionic acid [16]. In addition, *Coronilla* and *Indigofera* species have also been found to synthesize 3-nitropropionic acid [1,17]. When these plant materials were consumed by ruminant animals, it was rapidly hydrolyzed by ruminal β-glucosidase and esterase to liberate free nitropropanol or nitropropionic acid [1]. Both 3-nitropropanol glycosides and glucose conjugates of 3-nitropropionic acids were generally classified as nitrotoxins. Most ruminal microbes tolerate non-lethal concentrations of the nitrotoxins [11]. Under hydrolysis by ruminal microbes, nitropropanol and nitropropionic acid are further metabolized to 3-amino-1-propanol and β-alanine. For instance, a previous study noted that 87% of the 3-nitropropanol lost from incubation mixtures was recovered as 3-amino-1-propanol; conversely, β-alanine appeared to not be a terminal product and it was further metabolized to unidentified products [11].

Until now, reports concerning the reduction of industrially produced aliphatic nitrocompounds, such as NE, NEOH, and NPOH, are still rare. In the present study, an obvious and continuous reduction in NE, NEOH, and NPOH contents was observed, thus further confirming the presence of potential nitrocompound-degrading microbes within the rumen microbial community. The nitrocompound disappearance rate in the present study was ranked as: NE > NEOH > NPOH, suggesting that rumen microbes presented different metabolic capability to these nitrocompounds. Majak and Cheng [18] in an earlier study with both pure and mixed cultures confirmed that some rumen bacteria were indeed capable of anerobic degradation of naturally occurring 3-nitropropanol or 3-nitropropionic acid, and the ability to degrade these nitrocompounds and the rate at which they were degraded depended essentially on the particular strains of rumen bacteria (e.g., *Megasphaera elsdenii*, *Selenomonas ruminantium*). In subsequent studies, a new bacterium capable of metabolizing the naturally occurring nitrotoxins, 3-nitro-1-propanol and 3-nitro-1-propionate, was isolated from a ruminal population enriched for enhanced rates of nitrotoxin metabolism [19] and was accommodated as *Denitrobacterium detoxificans*, a new genus and species capable of degrading nitrocompounds into nitrite and then ammonia [20,21]. Although the present study was done with mixed cultures instead of pure culture, rumen microbes should have more or less similar degradation or metabolic action to NE, NEOH, and NPOH, like the 3-nitro-1-propanol and 3-nitro-1-propionate noted in the aforementioned studies. Of course, it is still necessary to conduct pure cultures in future studies to better understand why different degradation rates occurred for different nitrocompounds.

Due to the small accumulation of nitrite determined in the cultures incubated with 3-nitropropanol or 3-nitropropionic acid, Anderson et al. speculated that the cleavage of the nitrite moiety is not the primary metabolic pathway [11]. However, the phenomenon of small nitrite accumulation can be explained by the rapid reduction of nitrite to ammonia with ruminal microorganisms [22]. Nitrite contents, up to 0.09 to 0.10, 0.28 to 0.42, and 0.16 to 0.17 mM, were indeed detected in the present NE, NEOH, and NPOH groups after 3 h of incubation, respectively. However, the nitrite content was decreased to an undetectable value with the incubation time up to 96 h, which implied the capacity of ruminal microbes to reduce nitrite to ammonia [22].

### 3.3. Metabolic Fate of Nitrocompounds

As an important intermediate product, ammonia reflects not only its release from dietary protein degradation but also its consumption by ruminal microbes to synthesize microbial crude protein [23]. Ammonia content in the present nitrocompound-treated cultures was remarkably lower than that in the control; however, the accumulation of bacterial crude protein was significantly promoted with nitrocompound addition. Such results implicated that ammonia derived from nitrite could be efficiently utilized by ruminal microbes for microbial protein production. In addition, the unfavorably high NADH/NAD ratios suppress fermentation and subsequent deamination of reduced but not neutral or oxidized amino acids [24]. Thus, decreased accumulations of ammonia in the nitrocompound-treated cultures possibly also reflect increased intracellular accumulations of NADH.

Scott (1942) observed that acetaldehyde and nitrite appeared in the blood of nitroethane-dosed animals [25]. In addition, an earlier study by Angermaier and Simon [26] revealed the reduction of 2-nitroethanol to 2-aminoethanol through a nonspecific hydrogenase-ferredoxin system using clostridial whole-cell preparations. Therefore, it has been speculated that NE, NEOH, and NPOH could be degraded to their corresponding ethylamine, amino-ethanol, and 2-amino-1-propanol, respectively, and nitrite was formed. In the present study, a higher intermediate of nitrite occurred in NEOH in comparison with NPOH and NE while ammonia production was the lowest in NEOH. The different chemical structure and molecular weight of these nitrocompounds might lead to the distinct degradation rates. In terms of the chemical structure, both NE and NEOH are classified as primary aliphatic nitrocompounds, and NPOH is a secondary aliphatic nitrocompound. The primary and secondary aliphatic nitrocompounds differ in the number of acidic hydrogens on the carbon atom adjacent to the nitro group. For instance, secondary aliphatic NPOH has one hydrogen, but primary aliphatic NE and NEOH have two hydrogens. Therefore, the NPOH with only one acidic hydrogen has the lowest disappearance rate than NE and NEOH. Additionally, the molecular weight of NE is much lower than that of NEOH, which probably slowed down the nitrite formation from NE-treated cultures. Previous studies [27,28] noted that nitrite was able to bind the hemoglobin of red blood cells to form methemoglobin and further impede oxygen transportation. In this case, excessive accumulation of nitrite in the rumen ecosystem may be toxic to ruminants. An earlier study noted that blood methemoglobin at 3 g/dL did not affect the health of ruminants [29]. In the present study, nitrite accumulation was less than 2 mg/dL (1.5 mg/75 mL culture fluids) during the first 72 h, and it declined to zero at 96 h, implicating that the level of nitrite accumulation should not affect the health of the ruminant even if such a case occurred in vivo.

There were at least two sources of in vitro ruminal ammonia formation in the current nitrocompound-treated cultures: Dietary protein degradation and nitrite reduction. Generally, nitrite in the rumen can be metabolized rapidly to ammonia by nitrite-reducing microorganisms [27]. In response to the addition of nitrocompounds, the low ammonia N yield and high bacterial crude protein observed in the present study implicated that the NE, NEOH, and NPOH may promote microbial activities or the growth of rumen microbes.

## 4. Conclusions

Regardless of whatever substrate type was applied, both NE and NEOH in comparison with NPOH presented greater antimethanogenic activity via the shift of rumen fermentation (e.g., decreasing acetate:propionate ratio). Rumen microorganisms were able to degrade 91% to 92% of NE, 69% to 79% of NEOH, and 56% to 64% of NPOH, and the further metabolism of nitrite as intermediates into ammonia N may enhance the growth of rumen microbes or promote microbial activities.

## 5. Materials and Methods

All animal care and experimental operations described in this study were carried out following the Guidelines of the Institutional Animal Care and Use Committee of China Agricultural University (Beijing, China; CAU20171014-1).

### 5.1. Nitrocompounds

Liquid forms of nitroethane (NE), 2-nitroethanol (NEOH), and 2-nitroproponal (NPOH) were purchased commercially (Sigma-Aldrich, Inc., St. Louis, MO, USA) and stored at 4 °C. The analytical grades of NE, NEOH, and NPOH were 99%, 90%, and 98% as guaranteed, respectively.

### 5.2. Animals and Inoculant

Five lactating Holstein dairy cows (540 ± 25.3 kg body weight) were fitted with rumen cannulas (Type 2C; Bar Diamond Inc., Parma, ID, USA), and served as experimental animals. Animals with free access to water were routinely housed in a free stall and fed twice daily a total mixed ration. The ration (as fed per day) comprised 14 kg of maize silage, 3 kg of alfalfa hay, and 10 kg of concentrate, and it contained 1.74 Mcal/kg net energy for lactation, 140 g/kg of crude protein, 310 g/kg of neutral detergent fiber, and 190 g/kg of acid detergent fiber on a dry matter basis. On the day of starting the batch culture experiment, a rumen fluid sample after 2 h of morning feeding was collected from different compartments of the rumen via rumen cannulas of each cow and transferred to a pre-warmed (39 °C) thermal flask, which was filled with CO_2_ in advance. After arrival in the laboratory, rumen fluids were immediately strained through four layers of cheesecloth, mixed in equal portions, and used as inoculant of mixed rumen microorganisms.

### 5.3. Substrate Preparation

Alfalfa hay at the early-bloom stage was dried at 65 °C for 48 h in a forced air oven and then chopped and ground to pass through a 2-mm screen in a Kunjie mill (Beijing Kunjie Yucheng machine equipment Co., Ltd., Beijing, China). Then, the hay was mixed with maize meal by 1:4 and 4:1 (w/w) to prepare a low-forage (LF) and a high-forage HF) substrate, respectively.

### 5.4. Experimental Design

A completely randomized design was applied in in vitro batch cultures with the two substrates (HF vs. LF), and each substrate was treated with zero dose (control) and 10 mM of NE, NEOH, and NPOH. Sterile glass bottles (volume capacity of 120 mL) with Hungate stoppers and screw caps served as incubators. The experiment was conducted at 39 °C for 120 h and completed in 2 paralleled batches. In batch 1, 6 fermentations per treatment were arranged to determine the content of volatile fatty acid (VFA), ammonia N, bacterial crude protein (BCP), nitrite, and nitrocompound residue in culture fluids for an incubation time of 3, 6, 12, 24, 48, 72, 96, and 120 h. In batch 2, 4 fermentations per treatment were carried out for 120 h to collect fermentation gases for the determination of CH_4_, H_2_, and CO_2_ composition. Meanwhile, five substrate-free bottles without any nitrocompound addition served as blanks.

### 5.5. In Vitro Batch Culture and Sampling Procedure

In total, 500 mg of each substrate was weighed into 120-mL bottles in batch 1 and then filled with 49 mL of basal medium (pH 6.8) [30] and 25 mL of the freshly prepared ruminal fluid inoculant. Afterwards, 1 mL of the corresponding stock solution of each nitrocompound was added to the bottles for the NE, NEOH, and NPOH treatment group and 1 mL of distilled water for the controls. All the bottles were purged with pure N_2_ gas to remove headspace air, sealed with Hungate stoppers and screw caps, and continuously incubated at 39 °C in a temperature-controlled incubator. At each incubation time of 3, 6, 12, 24, 48, 72, 96, and 120 h, 1.0 mL of culture fluid per bottle were sampled with sterile syringes, and meanwhile 1.0 mL of fresh basal medium was injected into the bottle to compensate cultures. The samples were stored at −20 °C for later content analyses of VFA, ammonia N, BCP, nitrite, and residual nitrocompound.

Except the culture fluid sampling procedure in batch 1, four extra bottles per treatment in batch 2 were continuously connected with medical transfusion pipes to pre-emptied gasbags to collect whole fermentation gas end-products. After 120 h of incubation, 1-mL gas sample from the gasbags was removed for the subsequent determination of H_2_, CH_4_, and CO_2_.

### 5.6. Laboratory Analysis

Fluid aliquots were thawed and separated into 5 equal parts (200 μL each). An aliquot of culture fluids (200 μL) were prepared for quantification of nitrocompounds. The content of NE, NEOH, and NPOH was measured colorimetrically according to a modified method of Ochoa-García et al. [7] using a spectrophotometer (Ailaibao Medical Technology Co., Ltd., Jinan, China). Fluid samples were centrifuged at 10,000× *g* for 15 min. Supernatants or standards (50 μL) were then diluted with 2 mL of H_2_O, and then 100 μL of 0.65 M NaOH and 100 μL of diazotized p-nitroaniline were added. After 2 min of reaction, the absorbance was read at the 405-nm wavelength.

A second aliquot of culture fluids (200 μL) was prepared for determination of the nitrite content. Nitrite concentration was determined at the 540-nm wavelength by a colorimetrical method using the Griess Reagent System as described by Deng et al. [31]. Griess reagent consists of two parts p-amino benzene sulfonamide aqueous solution (1%) and N-1-napthylethylenediamine aqueous solution (0.1%).

Following the method of Pang et al. [32], ammonia N in the third aliquot of culture fluid was measured at the 637-nm wavelength colorimetrically using a microplate reader (RT-6500, Rayto Instruments, Shenzhen, China). Briefly, 100 μL of supernatant from each sample or standard was mixed with 45 μL of phenol reagent and 155 μL of hypochlorite reagents.

A fourth aliquot of culture fluid (200 μL) was transferred to a polypropylene tube for the bacterial crude protein (BCP) determination based on the method of Pang et al. [32] using the microplate reader as follows: 200 μL culture fluid samples were centrifuged at 25,000× *g* for 20 min. The sediment was alkaline hydrolyzed for 10 min at 95 °C, and then 50 μL of supernatant were mixed with 150 μL of Coomassie brilliant blue G-250 coloration solution and then measured under a wavelength of 595 nm.

At last, an aliquot of culture fluids (200 μL) was mixed with 0.3 mL of 25 mg/mL metaphosphoric acid solution for 30 min at 4 °C, and centrifuged at 10,000× *g* for 15 min at 4 °C. Total volatile fatty acids (VFAs) production in the supernatant was then determined using a gas chromatograph (GC522, Wufeng Instruments, Shanghai, China) equipped with a 15 m semicapillary column (Ø 0.53 mm) packed with Chromosorb 101, with pure N2 as the carrier gas at a column temperature of 120 °C [33].

For determination of the gas composition, a 1-mL gas sample was removed from the gasbags for the measurement of H_2_, CH_4,_ and CO_2_ following the method as described by Zhang and Yang [34]. The gas sample (1 mL) was injected to a gas chromatograph (GC522, Wufeng Instruments, Shanghai, China) packed with carbon porous beads (TDX-1) in a 2 m stainless steel column (2.0 mm inner diameter). The peaks of H_2_, CH_4_, and CO_2_ were identified by comparison with a standard of known composition [24].

### 5.7. Curve Fitting and Calculation

Nitrocompound, nitrite, ammonia N, and BCP concentrations (mM) at different incubation times (*C_t_*) were fitted to a one-compartment model [35] as Equations (1) and (2):

*C_t_* = *C*_0_ × *e*^(−*k* × *t*)^, (1)

*C*_t_ = *C*_0_ × *e*^(*k* × *t*)^, (2)

where *t* is the incubation time; *C*_0_ is the initial concentration at time *t* = 0; and *k* is the disappearance or accumulation rate (%/h) in the one-compartment model.

Calculation of the time until half of the initial nitrocompounds inclusion had disappeared (i.e., half time (T_1/2_)) is shown in Equation (3):

T_1/2_ = *log* (2/k) for 1-compartment model, (3)

where *k* is the same as described for Equation (4).

According to Anderson et al. [5], the fermentation efficiency (FE) was calculated as Equation (4):

FE = 100 × (0.62 × acetate + 1.09 × propionate + 0.78 × butyrate)/(acetate + propionate + butyrate),(4)

where acetate, propionate, and butyrate were expressed in molar proportions (mmol/mol) of the total VFA production.

### 5.8. Statistical Analysis

Data were analyzed using a 2 × 4 factorial arrangement in a completely randomized design. Two substrates (LF and HF) and four treatments (control, NE, NEOH, and NPOH), and interaction of treatment × substrate were included as fixed effects and an incubated glass bottle as a random effect were included in the model. The model was applied as Equation (5) using the PROC MIXED procedure of SAS 9.4 (Statistical Analysis for Windows, SAS Institute Inc., Cary, NC, USA):

Y_ij_ = μ + D_i_ + N_j_ + (D × N)_ij_ + B_k_ + ε_ijk_, (5)

where Y_ij_ is the dependent variable under examination, μ is the overall mean, D_i_ is the fixed effect of the substrates (I = 2), N_j_ is the fixed effect of nitrocompounds (j = control, NE, NEOH, and NPOH), D × N is the interaction effect between the nitrocompound treatment and substrate, B_k_ is the random effect of bottles, and εij is the error term. Least square means (LSMEANS) and standard errors of the means (SEM) were calculated using the LSMEANS statement of SAS. The orthogonal contrasts were used to assess the substrate effect. Overall differences among nitrocompound treatments were conducted using a general analysis of variance with a Tukey’s multiple comparison of means. Significance was declared at *p* < 0.05, and a tendency towards significance declared at *p* ≤ 0.10.

## Figures and Tables

**Table 1:** Effect of 10 mM addition of nitroethane (NE), 2-nitroalcohol (NEOH), and 2-nitro-1-propanol (NPOH) in culture fluids on the production of volatile fatty acids (VFAs, mmol per incubations) and their pattern of substrates with a high (HF) and low (LF) forage content incubated in batch cultures of mixed rumen microorganisms.

		Nitrocompounds ^1^					*p*-Value ^2^		
Items ^3^	Substrate	CTR	NE	NEOH	NPOH	SEM	S	N	S × N
total VFA, mmol	HF	6.8	6.7	6.6	6.8	0.15	< 0.01	0.63	0.79
	LF	7.5	7.4	7.2	7.2				
*Molar percentage of individual VFA, mol/100 mol*									
Acetate	HF	60.8 ^a^	59.7 ^b^	59.3 ^b^	59.2 ^b^	0.41	< 0.01	< 0.01	0.17
	LF	57.1 ^a^	54.1 ^b^	54.0 ^b^	54.1 ^b^				
Propionate	HF	23.0 ^c^	23.5 ^b^	24.2 ^a^	24.0 ^a^	0.18	< 0.01	< 0.01	0.07
	LF	24.6 ^c^	26.0 ^b^	27.3 ^a^	26.4 ^b^				
Butyrate	HF	9.4 ^b^	10.4 ^a^	10.1 ^a^	10.1 ^a^	0.12	< 0.01	< 0.01	0.13
	LF	11.7 ^b^	12.7 ^a^	12.4 ^a^	12.0 ^a^				
Valerate	HF	1.6	1.5	1.5	1.6	0.03	0.21	0.10	0.56
	LF	1.6	1.5	1.5	1.5				
BCVFA	HF	4.8	5.0	5.1	4.8	0.27	< 0.01	0.33	0.61
	LF	4.9	6.2	5.5	5.7				
Ace:Pro	HF	2.7 ^a^	2.6 ^b^	2.4 ^c^	2.5 ^bc^	0.03	< 0.01	< 0.01	0.15
	LF	2.3 ^a^	2.1 ^b^	2.0 ^b^	2.1 ^b^				
FE	HF	75.0 ^c^	75.6 ^b^	76.0 ^a^	75.8 ^ab^	0.13	< 0.01	< 0.01	0.08
	LF	76.4 ^b^	77.4 ^a^	77.7 ^a^	77.5 ^a^				

^a–c^ Means within a row without a common superscript letter differ at *p* < 0.05; ^1^ CTR, control group; NE, nitroethane; NEOH, 2-nitroethanol; NPOH, 2-nitropropional; ^2^ S = substrate; N = nitrocompounds; S × N = Interaction effect between substrate and nitrocompounds. ^3^ BCVFA, branch-chained volatile fatty acids, including iso-butyrate and iso-valerate; Ace: Pro, the ratio of acetate to propionate; FE, fermentation efficiency was calculated as 100 × (0.62 × acetate + 1.09 × propionate + 0.78 × butyrate)/(acetate + propionate + butyrate).

**Table 2:** Effect of 10 mM addition of nitroethane (NE), 2-nitroalcohol (NEOH), and 2-nitro-1-propanol (NPOH) addition in culture fluids on gas production and fermentation gas composition (% total gas production) of substrates with a high (HF) and low (LF) forage content incubated in batch cultures of mixed rumen microorganisms.

		Nitrocompounds ^1^					*p*-Value ^2^		
Items ^3^	Substrate	CTR	NE	NEOH	NPOH	SEM	S	N	S × N
GP_120_, mL/kg DM	HF	147 ^a^	128 ^b^	128 ^b^	145 ^a^	3.3	< 0.01	< 0.01	0.11
-	LF	217 ^a^	183 ^bc^	172 ^c^	187 ^b^	-	-	-	-
CH_4_, %	HF	21.0 ^a^	0.3 ^c^	0.5 ^c^	13.4 ^b^	0.05	< 0.01	< 0.01	0.23
-	LF	21.1 ^a^	0.2 ^c^	0.2 ^c^	8.6 ^b^	-	-	-	-
H_2_, %	HF	0.1 ^d^	5.8 ^a^	3.7 ^b^	1.2 ^c^	0.05	< 0.01	< 0.01	0.09
-	LF	0.1 ^c^	9.6 ^a^	8.8 ^ab^	6.9 ^b^	-	-	-	-
CO_2_, %	HF	78.9 ^c^	93.8 ^a^	95.8 ^a^	85.4 ^b^	0.79	0.01	< 0.01	0.10
-	LF	78.8 ^c^	90.3 ^a^	91.0 ^a^	84.5 ^b^	-	-	-	-

^a–d^ Means within a row without a common superscript letter differ at *p* < 0.05; ^1^ CTR, control group; NE, nitroethane; NEOH, 2-nitroethanol; NPOH, 2-nitropropional; ^2^ S = substrate; N = nitrocompounds; S × N = Interaction effect between substrate and nitrocompounds. ^3^ GP_120_, cumulative gas production at 120 h.

**Table 3:** The disappearance kinetics of nitrocompounds during in vitro ruminal fermentation of substrates with a high (HF) and low (LF) forage content incubated in batch cultures of mixed rumen microorganisms.

		Treatment ^1^					*p*-Value ^2^		
Items ^3^	Substrate	CTR	NE	NEOH	NPOH	SEM	S	N	S × N
*C*_0_, mM	HF	0	0.75	0.75	0.75	-	-	-	-
	LF	0	0.75	0.75	0.75	-	-	-	-
*k*, %/h	HF	0	2.8 ^a^	1.1 ^b^	0.8 ^b^	0.42	< 0.01	< 0.01	0.11
	LF	0	6.2 ^a^	1.2 ^b^	0.6 ^b^	-	-	-	-
*T*_1/2_, h	HF	0	4.6 ^c^	5.3 ^b^	5.5 ^a^	0.08	< 0.01	< 0.01	0.12
	LF	0	3.7 ^c^	5.1 ^b^	5.7 ^a^	-	-	-	-

^a–c^ Means within a row without a common superscript letter differ at *p* < 0.05; ^1^ CTR, control group; NE, nitroethane; NEOH, 2-nitroethanol; NPOH, 2-nitropropional; ^2^ S = Substrate; N = Nitrocompounds; S × N = Interaction effect between substrate and nitrocompounds; ^3^ Nitrocompounds concentration at different incubation times was fitted to the one-compartment model as follows: *C*_t_ = *C*_0_ × e^−k × t^; where t is the incubation time; *C*_0_ is the initial nitrocompounds does at time t = 0 when nitrocompounds were administered (10 mM); k (%/h) is the nitrocompounds disappearance rate; *T*_1/2_ = the time when half of *C*_0_ occurred (h).

**Table 4:** The disappearance kinetics of nitrite (NO_2_^−^) during in vitro ruminal fermentation of substrates with a high (HF) and low (LF) forage content incubated in batch cultures of mixed rumen microorganisms (from 3 to 120 h).

		Treatment ^1^					*p*-Value ^2^		
Items ^3^	Substrate	CTR	NE	NEOH	NPOH	SEM	S	N	S × N
*C*_3_, mM	HF	0	0.11 ^c^	0.40 ^a^	0.17 ^b^	0.012	< 0.01	< 0.01	0.13
	LF	0	0.09 ^c^	0.26 ^a^	0.15 ^b^				
*k*, %/h	HF	0	1.2 ^a^	1.0 ^b^	1.0 ^b^	0.02	0.83	< 0.01	0.12
	LF	0	1.1 ^a^	1.1 ^b^	0.9 ^b^				
*T*_1/2_, h	HF	0	5.1 ^b^	5.3 ^a^	5.3 ^a^	0.01	0.47	< 0.01	0.21
	LF	0	5.2 ^b^	5.2 ^b^	5.4 ^a^				

^a–c^ Means within a row without a common superscript letter differ at *p* < 0.05; ^1^ CTR, control group; NE, nitroethane; NEOH, 2-nitroethanol; NPOH, 2-nitropropional; ^2^ S = Substrate; N = Nitrocompounds; S × N = Interaction effect between substrate and nitrocompounds; ^3^ Nitrite concentration at different incubation times from 3 to 120 h was fitted to the one-compartment model as follows: *C*_t_ = *C*_3_ × e^−k × t^; where t is the incubation time; *C*_3_ is the maximum concentration of nitrite (NO_2_^−^) at time t = 3 h; k (%/h) is the nitrite disappearance rate; *T*_1/2_ = the time when half of *C*_3_ occurred (h).

**Table 5:** The accumulation kinetics of ammonia N during in vitro ruminal fermentation of substrates with a high (HF) and low (LF) forage content incubated in batch cultures of mixed rumen microorganisms.

		Treatment ^1^					*p*-Value ^2^		
Items ^3^	Substrate	CTR	NE	NEOH	NPOH	SEM	S	N	S × N
*A*, mM	HF	2.5 ^a^	2.3 ^b^	1.9 ^c^	2.4 ^a^	0.11	0.01	0.01	0.54
	LF	2.4 ^a^	1.8 ^b^	1.4 ^c^	1.7 ^b^				
*k*, %/h	HF	1.1 ^a^	1.0 ^a^	0.9 ^b^	1.0 ^a^	0.01	< 0.01	< 0.01	0.09
	LF	1.0 ^a^	0.8 ^b^	0.6 ^c^	0.7 ^b^				
*T*_1/2_, h	HF	5.2 ^c^	5.3 ^b^	5.5 ^a^	5.3 ^b^	0.02	< 0.01	< 0.01	0.12
	LF	5.3 ^c^	5.5 ^b^	5.8 ^a^	5.6 ^b^				

^a–c^ Means within a row without a common superscript letter differ at *p* < 0.05; ^1^ CTR, control group; NE, nitroethane; NEOH, 2-nitroethanol; NPOH, 2-nitropropional; ^2^ S = Substrate; N = Nitrocompounds; S × N = Interaction effect between substrate and nitrocompounds; ^3^ Ammonia N concentration at different incubation times was fitted to the 1-compartment model as follows: *C*_t_ = *C*_0_ × e^k × t^; where t is the incubation time; *C*_0_ = 0.70 mM, which is the initial ammonia N at time t = 0; k (%/h) is the ammonia N accumulation rate; A is the asymptotic ammonia N accumulation; *T*_1/2_ = the time when half of A occurred (h).

**Table 6:** The accumulation kinetics of bacterial crude protein (BCP) during in vitro ruminal fermentation of substrates with a high (HF) and low (LF) forage content incubated in batch cultures of mixed rumen microorganisms.

		Treatment ^1^					*p*-Value ^2^		
Items ^3^	Substrate	CTR	NE	NEOH	NPOH	SEM	S	N	S × N
*A*, g/L	HF	1.8 ^b^	2.2 ^a^	2.1 ^a^	2.2 ^a^	0.13	0.96	0.03	0.89
	LF	1.8 ^b^	2.3 ^a^	2.1 ^a^	2.1 ^a^				
*k, %/h*	HF	0.6 ^b^	0.8 ^a^	0.7 ^a^	0.8 ^a^	0.02	0.41	0.02	0.12
	LF	0.6 ^b^	0.8 ^a^	0.8 ^a^	0.8 ^a^				
*T*_1/2_, h	HF	5.8 ^a^	5.5 ^b^	5.6 ^b^	5.5 ^b^	0.02	0.09	< 0.01	0.11
	LF	5.7 ^a^	5.5 ^b^	5.6 ^b^	5.5 ^b^				

^a,b^ Means within a row without a common superscript letter differ at *p* < 0.05. ^1^ CTR, control group; NE, nitroethane; NEOH, 2-nitroethanol; NPOH, 2-nitropropional ^2^ S = Substrate; N = Nitrocompounds; S × N = Interaction effect between substrate and nitrocompounds. ^3^ BCP concentration at different incubation times was fitted to the one-compartment model as follows: *C*_t_ = *C*_0_ × e^k × t^; where t is the incubation time; *C*_0_ = 0.86 g/L, which is the initial BCP does at time t = 0; k (%/h) is the BCP accumulation rate; A is the asymptotic BCP accumulation; *T*_1/2_ = the time when half of A occurred (h).
